# Biochemical Characterization and Degradation Pattern of a Novel Endo-Type Bifunctional Alginate Lyase AlyA from Marine Bacterium *Isoptericola halotolerans*

**DOI:** 10.3390/md16080258

**Published:** 2018-07-31

**Authors:** Benwei Zhu, Limin Ning, Yucui Jiang, Lin Ge

**Affiliations:** 1 College of Food Science and Light Industry, Nanjing Tech University, Nanjing 211816, Jiangsu, China; 2 College of Medicine and Life Science, Nanjing University of Chinese Medicine, Nanjing 210023, Jiangsu, China; jiangyucuinju@163.com; 3 Technology Transfer Center, Nanjing Forest University, Nanjing 210037, Jiangsu, China; njfuelin@126.com

**Keywords:** *Isoptericola halotolerans*, bifunctional alginate lyase, oligosaccharides

## Abstract

Alginate lyases are important tools to prepare oligosaccharides with various physiological activities by degrading alginate. Particularly, the bifunctional alginate lyase can efficiently hydrolyze the polysaccharide into oligosaccharides. Herein, we cloned and identified a novel bifunctional alginate lyase, AlyA, with a high activity and broad substrate specificity from bacterium *Isoptericola halotolerans* NJ-05 for oligosaccharides preparation. For further applications in industry, the enzyme has been characterized and its action mode has been also elucidated. It exhibited the highest activity (7984.82 U/mg) at pH 7.5 and 55 °C. Additionally, it possessed a broad substrate specificity, showing high activities towards not only polyM (polyβ-d-mannuronate) (7658.63 U/mg), but also polyG (poly α-l-guluronate) (8643.29 U/mg). Furthermore, the *K_m_* value of AlyA towards polyG (3.2 mM) was lower than that towards sodium alginate (5.6 mM) and polyM (6.7 mM). TLC (Thin Layer Chromatography) and ESI-MS (Electrospray Ionization Mass Spectrometry) were used to study the action mode of the enzyme, showing that it can hydrolyze the substrates in an endolytic manner to release a series of oligosaccharides such as disaccharide, trisaccharide, and tetrasaccharide. This study provided extended insights into the substrate recognition and degrading pattern of the alginate lyases, with a broad substrate specificity.

## 1. Introduction

Alginate is the major component of the cell wall of brown algae [1]. It is a linear anionic heteropolysaccharide comprising of two uronic acids, α-l-guluronic (G) and β-d-mannuronic acid (M) [2]. The G and M subunits are covalently linked by 1,4-glycosidic linkage in three different types of blocks, homopolymeric α-l-guluronic acid (polyG), homopolymeric β-d-mannuronic acid (polyM), and heteropolymeric α-l-guluronic acid-β-d-mannuronic acid (polyMG) [3]. Because of the high viscosity, gelling properties, and versatile activities, alginate has been widely applied in food, chemical, and pharmaceutical industries [4,5,6]. However, the applications of this polysaccharide are still limited, and are subjected to the high molecular weight and poor solubility [7]. Thus, alginate oligosaccharide (AOS) has attracted more and more attention, as it retains the physiological functions and activities of alginate, but possessed smaller molecule weights and good bioavailability [8]. Yang et al. found that AOS can enhance the uptake of LDL (Low-Density Lipoprotein) by regulating the expression of LDLR (Low-Density Lipoprotein Receptor) and PCSK9 (Proprotein Convertase Subtilisin/kexin Type 9) [9]. Iwamoto et al. found that G8 (octaguluronic acid) and M7 (heptamannuronic acid) can induce RAW264.7 cells to produce cytokine furthest [10]. The similar effect of mannuronate oligomers have also been reported by Yamamoto et al. [11].

Alginate lyase can catalyze the alginate by the β-elimination and release unsaturated AOS with double bonds between C4 and C5 [12]. So far, hundreds of alginate lyases have been found in marine and terrestrial bacteria, marine mollusks, and algae, according to the CAZy database [13,14,15,16,17,18,19]. According to the substrate specificities, they can be classified into three types, polyM-specific lyases (EC 4.2.2.3), polyG-specific lyases (EC 4.2.2.11), and bifunctional lyases (EC 4.2.2.) [20]. Additionally, based on the sequence similarity, the enzymes are generally grouped into seven PL (Polysaccharide Lyases) families (PL-5, -6, -7, -14, -15, -17, and -18) [21]. Furthermore, alginate lyases can be grouped into endolytic and exolytic alginate lyases in terms of the mode of action [22]. Endolytic enzymes can cleave the glycosidic bonds inside the alginate with unsaturated oligosaccharides as the main products [23], while the exolytic ones degrade alginate into monomers [24]. So far, many endolytic alginate lyases have been widely used to produce AOS in food and nutraceutical industries [25]. Furthermore, they have also been used to elucidate the fine structures of alginate, prepare protoplast of brown algae [26,27], and treat cystic fibrosis, combined with antibiotics [28]. So far, many alginate lyases originating from marine microorganisms have been identified, gene-cloned, purified, and well characterized. However, few have been commercially used because of the poor substrate specificity and low activity [29,30,31]. In addition, there are few reports about the bifunctional alginate lyase with broad substrate specificity. Thus, it will be of great importance to explore the novel enzymes with excellent characteristics, such as a high activity and broad substrate specificity.

In this work, a new bifunctional alginate lyase with a high activity and broad substrate specificity has been cloned from *Isoptericola halotolerans* NJ-05, followed by being identified and characterized. The enzymatic kinetics were further characterized and the degrading products were also analyzed, which suggested it a good candidate to expand the applications of alginate lyases in food and nutraceutical industries.

## 2. Results and Discussions

### 2.1. Screening and Identification of Strain NJ-05

**Figure 1:**
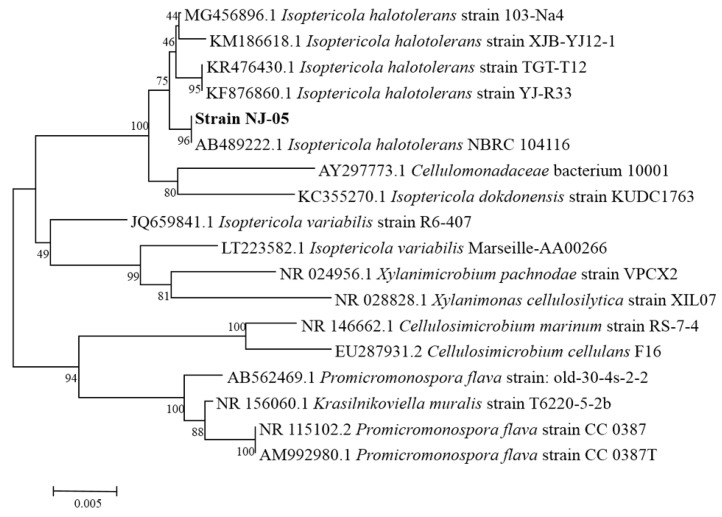
The phylogenetic analysis of strain NJ-05 and other similar strains. The phylogenetic tree was constructed by MEGA 6.0 (https://www.megasoftware.net/), based on the 16S rRNA gene sequences of strain NJ-05 and other known species.

According to the screening results by the plates and activity assay, the strain NJ-05 from the rotten brown algae obtained from the East China Sea showed the maximal activity of alginate lyase and then was identified for further investigation. The 16S rRNA sequence of the strain was then sequenced (GeneBank No. MH390700) and according to the phylogenetic analysis of the 16S rRNA sequence, the strain was assigned to the genus *Isoptericola* and named *Isoptericola halotolerans* NJ-05 (Figure 1).

### 2.2. Sequence Analysis of Alginate Lyase

The genome of *Isoptericola halotolerans* NJ-05 was firstly sequenced. After the annotation of the genomic information, several putative alginate lyase genes were found in a gene cluster for the alginate metabolism and the AlyA was identified as a putative alginate lyase by multiple sequences alignment. Then, the gene AlyA was cloned from the genome of *Isoptericola halotolerans* NJ-05 and sequenced (GeneBank No. MH390701). It can be observed that the open reading frame (ORF) consists of 771 bp, which encodes a putative alginate lyase with 256 amino acids, including a signal peptide with 32 amino acids (Supplementary Materials). According to the conserved domain analysis, AlyA possesses only a C-terminal catalytic domain consisting of 213 amino acids (Thr43-Glu255).

**Figure 2:**
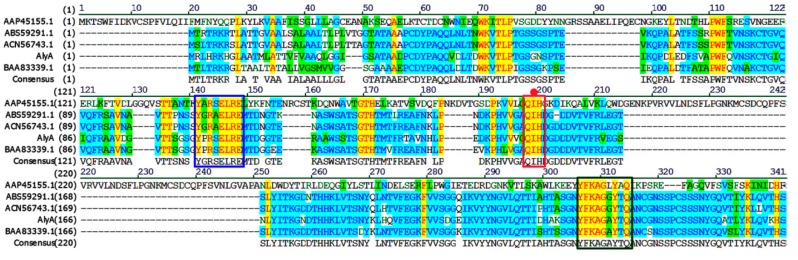
Multiple sequences alignments of alginate lyase (AlyA) and related alginate lyases of the PL7 family. The conserved regions and identical residues were highlighted with bands and red star, respectively.

**Figure 3:**
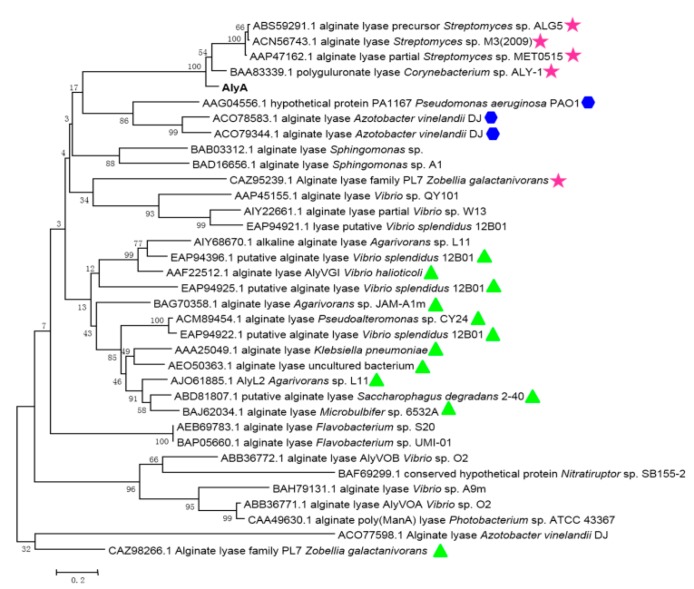
Phylogenetic analysis of AlyA and other alginate lyases of PL7. The phylogenetic tree was generated by the neighbor-joining method using MEGA 6.0 software (https://www.megasoftware.net/). The species names are indicated along with the accession number of the corresponding alginate lyase sequence. Bootstrap values of 1000 trials are presented in the branching points. The scale bar indicating ten nucleotide substitutions per 100 nucleotides is indicated at the bottom. The alginate lyases of subfamily 1, 3, and 5 were marked with a blue hexagon, red pentacles, and green triangles, respectively.

The sequence alignment of AlyA and other alginate lyases of the PL7 family are shown in Figure 2. It can be observed that AlyA shares the highest identity, of 79%, with alginate lyase (ACN56743.1) from *Streptomyces* sp. M3 [32] and exhibited an identity of 67% with alginate lyase (BAA83339.1) from *Corynebacterium* sp. ALY-1 [33]. Additionally, it also contains the conserved regions “PRT/V/SELRE”, “YFKA/VGN/VY”, and “QIH”, which are related to the substrate combination and catalytic activity (Figure 2). Thus, AlyA is a member of the PL7 family. Moreover, as the alginate lyases of PL7 family are further grouped into five families (1–5) according to the amino acid sequence homology, the phylogenetic tree was constructed by comparing the sequence homology of AlyA with the alginate lyases from a different subfamily to determine the subfamily of AlyA (Figure 3). It can be observed that the AlyA clusters represent the enzymes of subfamily 3, indicating that AlyA is the member of the subfamily 3 alginate lyases.

### 2.3. Expression and Purification of AlyA

**Figure 4:**
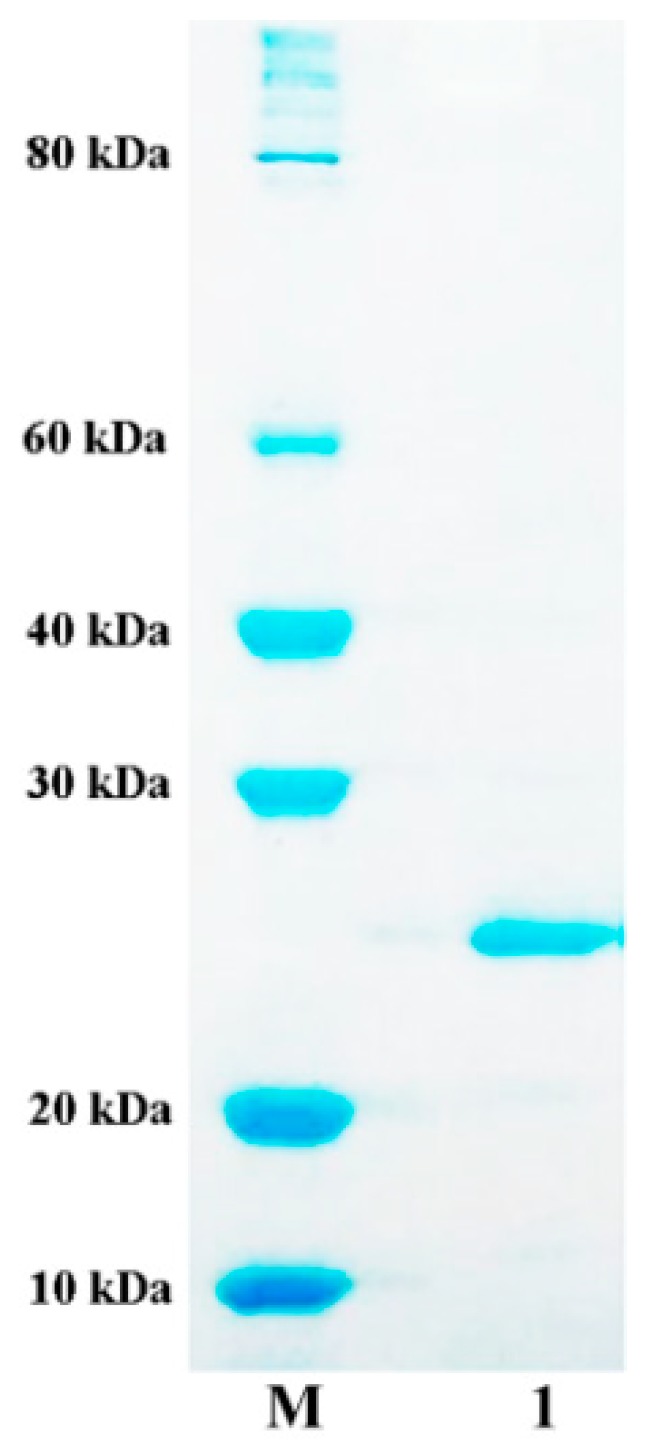
The sodium dodecyl sulfate polyacrylamide gel electrophoresis (SDS-PAGE) analysis of purified alginate lyase, AlyA. Lane M—the protein molecular weight standard; lane 1—the purified AlyA.

For a better characterization, the recombinant protein was expressed by firstly inserting the AlyA gene into the pET-21a (+) vector, and then being purified by Ni-NTA(Ni- Nitrilotriacetic acid) column (The summary of purification was shown in Table S1). The molecular mass of the recombinant protein with (His)_6_-tag is calculated to be 25.32 kDa, which is smaller than two other bifunctional alginate lyases, the Aly-SJ02 of *Pseudoalteromonas* sp. SM0524 with a bigger molecular mass of 32 kDa [34] and the Aly202 of *Alteromonas* sp. No. 272 with a similar molecular mass of 33.9 kDa [35]. The recombinant AlyA was further purified by Ni-NTA Sepharose affinity chromatography and analyzed by sodium dodecyl sulfate polyacrylamide gel electrophoresis (SDS-PAGE) (Figure 4). A single band of purified AlyA was observed at the gel, and it can be further used for downstream biochemical characterization.

### 2.4. Substrate Specifity and Enzymatic Kinetics of the Enzyme

As shown in Table 1, AlyA possessed a broad substrate specificity, showing a higher activity towards sodium alginate (7984.82 U/mg) and polyG (8643.29 U/mg), and a lower activity towards polyM (7658.63 U/mg). Thus, it can be concluded that AlyA is a bifunctional alginate lyase. Compared with another bifunctional alginate lyase, Aly-SJ02, which displayed almost the same activity towards sodium alginate (4802.7 U/mg) and polyM (4153.8 U/mg), but lower activity towards polyG (3073.7 U/mg) [34], it exhibited a much higher activity to all three of the substrates. Similarly, alginate substrates with various MM (Manuronate-Manuronate) and GG (Guluronate- Guluronate) ratios were used to further confirm the substrate affinity and substrate specificity of FAly and SALy. FAly prefer to degrade polyM, as the initial rates towards polyM are almost double that towards polyG, while SALy performed slightly better on polyG than on polyM [36].

The enzymatic kinetics of AlyA towards sodium alginate, polyM, and polyG were also studied according to the hyperbolic regression analysis. As shown in Table 1, the *K_m_* values of AlyA towards sodium alginate, polyM, and polyG were 5.6 mM, 6.7 mM, and 3.2 mM, respectively. Thus, it has higher affinity towards sodium alginate and polyG than that to polyM. The *k_cat_*/*K_m_* values of AlyA towards sodium alginate (8.20 mM^−1^∙s^−1^), polyG (7.56 mM^−1^∙s^−1^), and polyM (4.02 mM^−1^·s^−1^) were also calculated, indicating that the enzyme exhibited a higher catalytic efficiency towards sodium alginate and polyG than to polyM. Therefore, AlyA prefers the G block or MG block to the M block as the fit substrate, which is consistent with the substrate specificity of AlyA. It can be reasonable, as the conserved residue “I” in AlyA can recognize the polyG block or MG blocks [20]. As to the calculation of the kinetics data, the *K_m_* and *V_max_* values of some alginate lyases were determined by double-reciprocal plots of Lineweaver and Burk. For instance, Algb from *Vibrio* sp. W13 showed lower *K_m_* values toward alginate (0.67 mg/mL) polyG (1.04 mg/mL) than that to polyG (6.90 mg/mL) [17], and Alg-S5 from *Exiguobacterium* sp. Alg-S5 exhibited a high affinity towards the alginate with a lower *K_m_* value of 0.91 mg/mL [37]. To conveniently calculate the *k_cat_* and *k_cat_*/*K_m_* values, we referred the calculation method in the literature [38], and described the details of the calculation of the molar concentration and molecular weight in our recent publications [23,30,39,40,41].

### 2.5. Biochemical Characterization of AlyA

**Figure 5:**
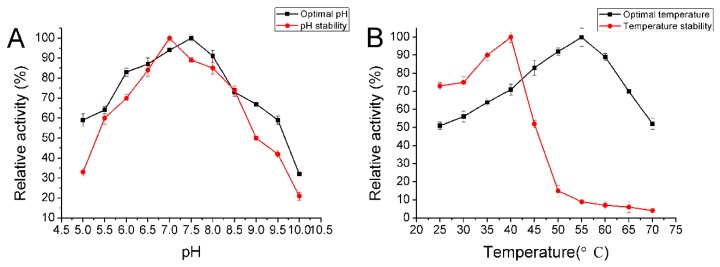
The biochemical characteristics of AlyA. (**A**) The optimal pH and the pH stability of AlyA. (**B**) The optimal temperature and the thermal stability of AlyA. The assay was then incubated at 40 °C for 10 min. Each value represents the mean of three replicates ± standard deviation.

AlyA was further characterized biochemically. The optimal pH for the enzyme activity was 7.5 and retained more than 60% activity after being incubated at a broad pH range of pH 5.5–9.0 for 24 h (Figure 5A). Additionally, the enzyme was mostly stable at pH 7.0. As previously reported, the alginate lyases of the PL7 family were active at a neutral pH. The Alg7D from *Saccharophagus degradans,* AlgNJ-04 from *Vibrio* sp. NJ-04, FsAlgA from *Flammeovirga* sp. NJ-04, and AlyA1 from *Zobellia galactanivorans* all showed their maximal activity at pH 7.0 [13,39,41,42]. However, they usually show a high activity in a narrow pH range, and exhibit instability under alkaline conditions. For instance, the AlySJ-02 from *Pseudoalteromonas* sp. SM0524 exhibited its maximal activity at pH 8.0 and retained its stability between pH 7.0–9.0 [34]. The AlyIH from *Isoptericola halotolerans* CGMCC5336 showed the highest activity at pH 7.0 and was stable at pH 7.0–8.0 [43]. Similarly, the A9m from *Vibrio* sp. A9mT exhibited its maximal activity at pH 7.5 and could maintain its stability between pH 7.0–9.0 [44].

AlyA showed maximum activity at 55 °C and was stable below 40 °C (Figure 5B). This enzyme possessed an approximately 80% activity after incubation at 40 °C for 30 min, and was gradually inactivated as the temperature increased. Similarly, most of the characterized enzymes of the PL7 family showing maximal activity around 30–40 °C (Table 2). For example, the AlgNJ-04 from *Vibrio* sp. NJ-04 [39] and AlgNJU-03 from *Vibrio* sp. NJU-03 [40] both possessed the optimal temperature of 40 °C. AlgC-PL7 from *Cobetia* sp. NAP1, OalY1 from Halomonas sp. QY114, and an alginate lyase from an unknown marine bacterium showed their maximal temperature at 45 °C [45,46,47]. Remarkably, Aly-SJ02 from *Pseudoalteromonas* sp. SM0524, FsAlgA from *Flammeovirga* sp. NJ-04, Alg7D from *Saccharophagus degradans*, AlgMsp from *Microbulbifer* sp. 6532A, and OalY2 from *Halomonas* sp. QY114 all had a higher optimal temperature of 50 °C [34,41,47]. Therefore, the AlyA possessed a great potential for industrial applications, due to the higher optimal temperature.

**Figure 6:**
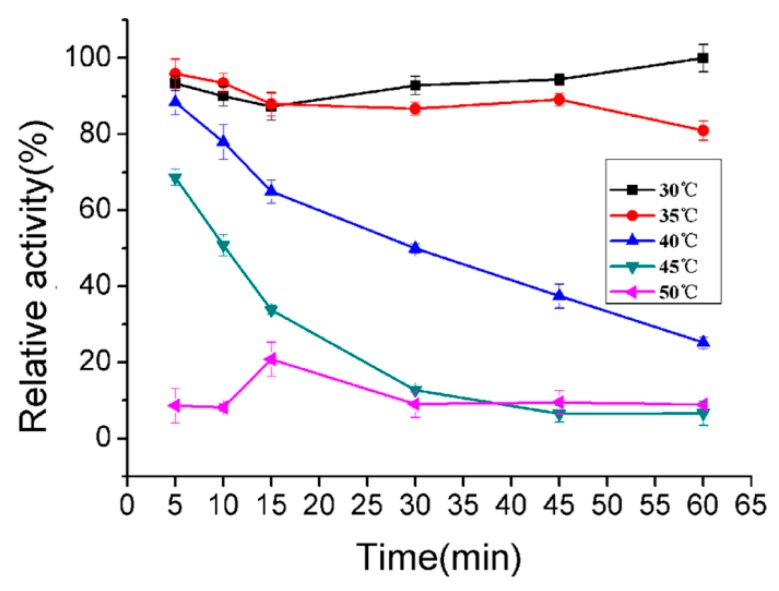
The thermal degeneration curve of AlyA. The maximal activity of the treated enzyme was regarded as 100% and the other relative activity was determined. Each value represents the mean of three replicates ± standard deviation.

The thermal stability of AlyA were further investigated by thermal-induction, as shown in Figure 6. The enzyme could retain almost 80% of its maximal activity after been incubated at 35 °C for 60 min. Similarly, the OalY1 and OalY2 from *Halomonas* sp. QY114 retained about 80% of the initial activities after incubation at 30 °C for 1 h [47]. While the AlgC-PL7 maintained approximately 80% of it activity at 70 °C [45]. As with the alginate lyase from the unknown marine bacterium, its activity remained without a noticeable loss up to 70 °C, with a monotonic decrease beyond this temperature [46].

The effect of metal ions on the activity of AlyA is shown in Table 3. It can be observed that Na^+^, Mg^2+^, and Ca^2+^ could greatly enhance the activity of the enzyme, while some divalent ions, such as Co^2+^, Cu^2+^, and Fe^3+^, inhibited the activity. Similarly, Ca^2+^ can activate AlyA from *Pseudomonas* sp. E03 [48], the AlyA from *Azotobacter chroococcum* 4A1M [49], and ALYII from *Pseudomonas* sp. OS-ALG-9 [50], as it could enhance the substrate-binding ability of the enzyme.

**Figure 7:**
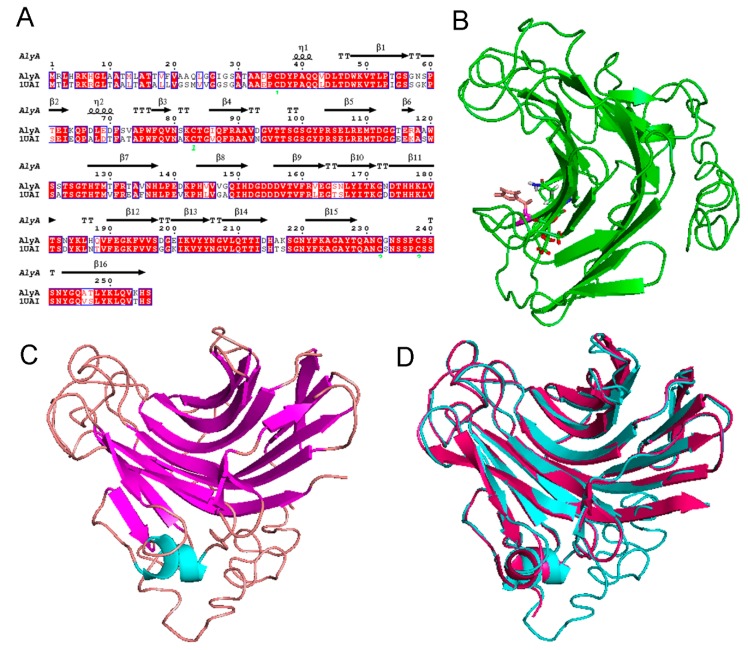
(**A**) The sequence alignment of AlyA and AlyPG from *Corynebacterium* sp. ALY-1, (**B**) the modeling structure of AlyA, (**C**) the structural comparison of AlyA (marked with red) and AlyPG (marked with blue) from *Corynebacterium* sp. ALY-1 (PDB ID: 1UAI), and (**D**) the key residuals for substrate reorganization of AlyA.

The sequence alignment of AlyA and AlyPG from *Corynebacterium* sp. ALY-1 was constructed by CLSTALW (http://www.clustal.org/) (as shown in Figure 7A). The three-dimensional model of the AlyA was constructed based on the homologues structure of the alginate lyase, AlyPG, of *Corynebacterium* sp. ALY-1 (PDB ID: 1UAI), using PHYRE2 (http://www.sbg.bio.ic.ac.uk/phyre2/html/page.cgi?id=index). As shown in Figure 7C, the overall structure of the AlyA was predicted to fold as a β-sandwich jelly roll formed, using two anti-parallel β sheets. The outer convex sheet includes five β-strands, and the inner concave sheet contains seven β-strands forming a groove that harbors the catalytic active site. In order to identify the key residues for substrate recognition, the structural alignment of AlyA and AlyPG was analyzed (Figure 7C). The two enzymes share a similar structure, and the residues Q159, H161, and R119 essential for substrate reorganization form hydrogen bonds with the carboxyl groups in subsites +1, +2, and +3, respectively (Figure 7A,D).

**Figure 8:**
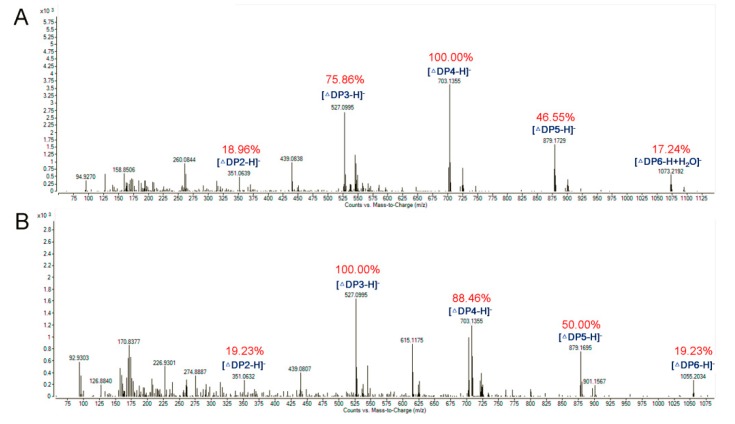
ESI-MS analysis of the degradation products of AlyA with (**A**) polyM and (**B**) polyG as substrate. The reaction mixtures (800 μL) containing 1 μg purified enzyme and 2 mg substrates (polyG and polyM) were incubated at 30 °C for 48 h.

The ESI-MS analysis of the degradation products is shown in Figure 8; disaccharides, trisaccharides, and tetrasaccharides account for a major fraction of the hydrolysates of two kinds of substrates. Thus, AlyA can be a potential tool to produce oligosaccharides with lower Dps by hydrolyzing sodium alginate. So far, most of the characterized alginate lyases of the PL7 family are endolytic enzymes, which can release oligosaccharides with a low Dp of 2–5 as the main products. Interestingly, AlyA5 from *Zobellia galactanivorans* can release disaccharides in an exolytic manner [42].

## 3. Materials and Methods

### 3.1. Materials

Sodium alginate derived from brown seaweed was purchased from Sigma (St. Louis, MO, USA, M/G ratio: 77/23). PolyM (purity: about 99%) and polyG (purity: about 99%) were purchased from Qingdao BZ Oligo Biotech Co., Ltd. (Qingdao, China). Other chemicals and reagents used in this study were of analytical grade.

### 3.2. Screening and Identification of Strain NJ-05

The decaying seaweed samples were collected from the coast of the East China Sea (123°11’ E, 25°10’ N), washed by sterilized sea water, and then spread on sodium alginate-agar plates (modified marine broth 2216 medium containing 5 g/L (NH_4_)_2_SO_4_, 19.45 g/L NaCl, 12.6 g/L MgCl_2_·6H_2_O, 6.64 g/L MgSO_4_·7H_2_O, 0.55 g/L KCl, 0.16 g/L NaHCO_3_, 1 g/L ferric citrate, 15 g/L agar, and 10 g/L sodium alginate). The strains with ability to produce alginate lyase were screened according to the procedures previously reported [30]. Furthermore, the activity of alginate lyase was determined by 3, 5-dinitrosalicylic acid (DNS) colorimetry [51]. Among the isolates, the most active strain NJ-05 was selected and identified for further studies by the alignment of the 16S rRNA sequence. A phylogenetic tree was constructed using CLUSTAL X (http://www.clustal.org/) and MEGA 6.0 (https://www.megasoftware.net/) through the neighbor-joining method.

### 3.3. Cloning, Expression, and Purification of the Alginate Lyase

The strain *Isoptericola halotolerans* NJ-05 was genome-sequenced and its genomic information was analyzed. Alginate utilization loci has been found and there are three putative alginate lyases within the cluster. Therefore, the primers for cloning AlyA were designed on the basis of the sequence of the putative alginate lyase gene sequence (No.chr_1816) within the genome of *Isoptericola halotolerans* NJ-05. The AlyA gene was amplified with primers designed as follows: the forward primer, 5′-ATGCGCCTGCATCGCAAAC-3′, and the reverse primer, 5′-GCTATGTTTCACCTGCAGTT-3′, from the genomic DNA of *Isoptericola halotolerans* NJ-05.

The alginate lyase gene was then subcloned into the pET-21a (+) expression vector for heterologously expression with restriction sites of *Nde*I and *Xho*I. The recombinant *E. coli* BL21 (DE3) harboring the pET-21a (+)/AlyA was cultured in an LB medium (containing 100 μg ampicillin/mL) for 2–3 h with shaking at 200 rpm and 37 °C up to an OD600 of 0.4–0.6. The cells were induced by adding 0.1 mM IPTG and then cultured at 20 °C for 30 h. The AlyA was purified by an NTA-column, as previously described [44]. The active fraction was collected, desalted, and then analyzed by 12% sodium dodecyl sulfate polyacrylamide gel electrophoresis (SDS-PAGE).

### 3.4. Enzyme Activity Assay

The assay including the purified enzyme (0.1 mL, 1.78 mg/mL) and 0.9 mL Tris-HCl (20 mM, pH 8.0, 1% sodium alginate) was then incubated at 40 °C for 10 min, as previously described [30]. The reaction was stopped by heating in boiling water for 10 min. The enzyme activity was then assayed by measuring the increased absorbance at 235 nm, and the enzymatic activity (one unit) was defined as the amount of enzyme required to increase the absorbance at 235 nm by 0.01 per min [38].

### 3.5. Substrate Specificity and Kinetic Measurement of Alginate Lyase

The assays of the enzyme activity for sodium alginate, polyM, and polyG were defined as described previously for investigating the substrate specificity. The kinetic parameters of the purified enzyme toward different substrates, including sodium alginate, polyM, and polyG, were determined by measuring the enzyme activity with substrates at different concentrations (0.1–8.0 mg/mL). The concentrations of the different substrates was calculated according to the method previously described [38]. As alginate consists of random combinations of mannuronic acid and guluronic acid residues with the same molecular weight (MW), substrate molarity can be calculated using the MW of 176 g/mol for each monomer of uronic acid in the polymer (i.e., 194 g/mol monomer MW − 18 g/mol for the loss of H_2_O during polymerization). The concentrations of the product were determined from the increase in absorbance at 235 nm using the extinction coefficient of 6150 M^−1^ cm^−1^. The velocity (V) at the tested substrate concentration was calculated as follows: V (mol/s) = (milliAU/min × min/60 s × AU/1000 milli AU × 1 cm)/(6150 M^−1^ cm^−1^) × (2 × 10^−4^ L). In addition, the *K_m_* and *V_max_* values were calculated by hyperbolic regression analysis, as described previously [38]. Additionally, the turnover number (*k_cat_*) of the enzyme was calculated by the ration of *V_max_* versus the enzyme concentration ([*E*]).

### 3.6. Biochemical Characterization of AlyA

The effects of temperatures (25–70 °C) on the purified enzyme were investigated at pH 9.0. The thermal stability of the enzyme was determined at pH 7.5 under the assay conditions described previously after incubating the purified enzyme at 25–70 °C for 30 min. In addition, the thermally-induced denaturation was also investigated by incubating the enzyme at 30–50 °C for 0–60 min at pH 7.5. Moreover, the effects of pH on the enzyme activity were evaluated by incubating the purified enzyme in buffers with different pHs (5.0–10.0) at 40 °C under the assay conditions described previously. The pH stability depended on the residual activity after the enzyme was incubated in buffers with different pHs (5.0–10.0) for 24 h, and then the residual activity was determined at 40 °C under the assay conditions. Meanwhile, the buffers with different pHs were used for phosphate–citrate (pH 5.0), NaH_2_PO_4_–Na_2_HPO_4_ (pH 6.0–8.0), Tris–HCl (pH 7.0–9.0), and glycine–NaOH (pH 10.0).

The influence of metal ions on the activity was performed by incubating the purified enzyme with various metal compounds at a concentration of 1 mM at 4 °C for 24 h. Then, the activity was measured under standard test conditions. The reaction mixture without any metal ion was taken as the control.

### 3.7. Molecular Modeling and Structural Alignment

The three-dimensional structure of AlyA was constructed using Protein Homology/analogY Recognition Engine V 2.0 (http://www.sbg.bio.ic.ac.uk/phyre2/html/page.cgi?id=index), on the basis of the homologues of the known structure (alginate lyase AlyA of *Corynebacterium* sp. ALY-1 (PDB ID: 1UAI) [52]. PyMOL (http://www.pymol.org) was used to visualize and analyze the modeled structure and to construct graphical presentations and illustrative figures.

### 3.8. ESI-MS Analysis of the Degradation Products of AlyA

For investigating the degradation pattern of AlyA, the reaction mixtures (800 μL) with pH 7.5 containing 1 μg purified enzyme and 2 mg substrates (polyG and polyM, the average Dp of the two substrates are about 40) were incubated at 30 °C for 0–48 h. After incubation, the mixture solutions were boiled for 10 min and then centrifuged at 12,000 rpm for 10 min to remove the unsolved materials. The hydrolysates were loaded onto a carbograph column (Alltech, Grace Davison Discovery Sciences, United Kingdom) to remove the salts after removing the proteins, and then concentrated, dried, and re-dissolved in 1 mL methanol. The supernatant (2 μL) was loop-injected to an LTQ XL linear ion trap mass spectrometer (Thermo Fisher Scientific, Waltham, MA, USA) after centrifugation, to further determine the composition of the products. The oligosaccharides were detected in a negative-ion mode using the following settings, previously described, with the scanning mass range 150–2000 *m*/*z* [30].

## 4. Conclusions

In this work, an alginate lyase-producing bacterium was isolated and identified to be *Isoptericola halotolerans* NJ-05. A novel bifunctional alginate lyase, AlyA, with high activity and broad substrate specificity was cloned and characterized. It exhibited the highest activity (7984.82 U/mg) at pH 7.5 and 55 °C. Additionally, it possessed broad substrate specificity, showing high activities towards not only polyM but also polyG. Furthermore, the *K_m_* value of AlyA towards polyG (3.2 mM) was lower than that towards sodium alginate (5.6 mM) and polyM (6.7 mM). The TLC and ESI-MS analyses indicated that it can hydrolyze the substrates in an endolytic manner to release a series of oligosaccharides such as disaccharide, trisaccharide, and tetrasaccharide. This study provided extended insights into the substrate recognition and degrading pattern of alginate lyases with broad substrate specificity.

## Figures and Tables

**Table 1:** The substrate specificity and enzymatic kinetics of alginate lyase (AlyA) towards various substrates.

Parameters	Sodium Alginate	PolyG	PolyM
Specific activity (U/mg)	7984.82	8643.29	7658.63
*K_m_* (mM)	5.6	3.2	6.7
*V_max_* (nmol/s)	3.22	1.74	1.89
*k_cat_* (s^−1^)	45.92	24.82	26.95
*k_cat_/K_m_* (s^−1^·mM^−1^)	8.20	7.56	4.02

**Table 2:** Comparison of biochemical properties of AlyA and partial enzymes of PL7 family.

Enzyme	Specificity	*K_m_*, *V_max_*	Optimal Temperature and pH	Products (Dp)	Reference
AlyA	Bifunctional	5.6 mM, 3.22 nmol/s	55 °C, 7.5	2–5	This study
Algb	polyG > polyM	0.67 mg/mL, 473.93 U/mg	30 °C, 8.0	2–5	[17]
FsAlgA	polyG > polyM	0.48 mM, 0.19 nmol/s	50 °C, 7.0	2–5	[41]
Alg7D	polyM > polyG	3.0 mg/mL, 6.2 U/mg	50 °C, 7.0	2–5	[13]
AlgMsp	polyG > polyM	3.4 mM, 57 pmol/s	50 °C, 8.0	2–5	[38]
AlgNJ-04	polyG > polyM	0.49 mM, 72 pmol/s	40 °C, 7.0	2–5	[41]
AlgNJU-03	polyG > polyM	8.5 mM, 1.67 nmol/s	30 °C, 7.0	2–4	[40]
AlgC-PL7	Bifunctional	-	55 °C, 8.0	1	[45]
A9m	polyG > polyM	-	30 °C, 7.5	-	[44]

**Table 3:** The effect of metal ions on activity of AlyA.

Reagent	Relative Activity (%)
Control	100 ± 0.5
Na^+^(100 mM)	126 ± 2.2
Na^+^(300 mM)	180 ± 3.1
Na^+^(500 mM)	203 ± 4.6
Na^+^(700 mM)	136 ± 2.9
Na^+^(900 mM)	89 ± 7.9
Zn^2+^	91 ± 2.3
Cu^2+^	65 ± 3.2
Mn^2+^	94 ± 2.1
Co^2+^	75 ± 3.4
Ca^2+^	174 ± 1.3
Ca^2+^(10 mM)	135 ± 5.7
Fe^3+^	88 ± 2.1
Mg^2+^	168 ± 2.7
Mg^2+^(10 mM)	119 ± 2.9
Mg^2+^(50 mM)	101 ± 3.2
Ni^2+^	87 ± 1.5

## Supplementary Materials

The following are available online at http://www.mdpi.com/1660-3397/16/8/258/s1, The nucleotide and protein sequence of AlyA and Table S1. Purification of the recombinant AlyA.
