# Supplementary material for: Biochemical Characterization and Degradation Pattern of a Novel Endo-Type Bifunctional Alginate Lyase AlyA from Marine Bacterium Isoptericola halotolerans
Source: Mar Drugs. 2018 Jul 31;16(8):258. doi: 10.3390/md16080258 (PMC6117692; doi:10.3390/md16080258)
Supplement: Supplementary file 1 [file marinedrugs-16-00258-s001.zip › Supplementary Materials/Supplementary Materials.docx]

**Supplementary Materials**

**The DNA and amino acids sequences of AlyA**

**MRLHRKHGLAATMLATTVFVAAQLGGIGSATAADPCDYPAQQVDLTDWKVTLPTGSGNSP**

**TEIKQPDLEDFSVAPWFQVNSKCTGIQFRAAVDGVTTSGSGYPRSELREMTDGGTERAAW**

**SSTSGTHTMTFRTAVNHLPEDKPHVVVGQIHDGDDDVTVFRVEGSNLYITKGNDTHHKLV**

**TSNYKLHQVFEGKFVVSDGEIKVYYNGVLQTTIDHAKSGNYFKAGAYTQANCGNSSPCSS**

**SNYGQATLYKLQVKHS**

**ATGCGCCTGCATCGCAAACATGGCCTGGCGGCGACCATGCTGGCGACCACCGTGTTTGTGGCGGCGCAGCTGGGCGGCATTGGCAGCGCGACCGCGGCGGATCCGTGCGATTATCCGGCGCAGCAGGTGGATCTGACCGATTGGAAAGTGACCCTGCCGACCGGCAGCGGCAACAGCCCGACCGAAATTAAACAGCCGGATCTGGAAGATTTTAGCGTGGCGCCGTGGTTTCAGGTGAACAGCAAATGCACCGGCATTCAGTTTCGCGCGGCGGTGGATGGCGTGACCACCAGCGGCAGCGGCTATCCGCGCAGCGAACTGCGCGAAATGACCGATGGCGGCACCGAACGCGCGGCGTGGAGCAGCACCAGCGGCACCCATACCATGACCTTTCGCACCGCGGTGAACCATCTGCCGGAAGATAAACCGCATGTGGTGGTGGGCCAGATTCATGATGGCGATGATGATGTGACCGTGTTTCGCGTGGAAGGCAGCAACCTGTATATTACCAAAGGCAACGATACCCATCATAAACTGGTGACCAGCAACTATAAACTGCATCAGGTGTTTGAAGGCAAATTTGTGGTGAGCGATGGCGAAATTAAAGTGTATTATAACGGCGTGCTGCAGACCACCATTGATCATGCGAAAAGCGGCAACTATTTTAAAGCGGGCGCGTATACCCAGGCGAACTGCGGCAACAGCAGCCCGTGCAGCAGCAGCAACTATGGCCAGGCGACCCTGTATAAACTGCAGGTGAAACATAGCTAA**

**Table S1.** Purification of the recombinant AlyA.

| **Purification step** | **Total protein (mg)** | **Volume (mL)** | **Total activity(U)** | **Specific activity (U/mg)** | **Purification (fold)** | **Yield (%)** |
| --- | --- | --- | --- | --- | --- | --- |
| Crude enzyme | 15.38 | 20 | 24730.76 | 1607.98 | 1.00 | 100 |
| Ni-NTA column | 2.23 | 5 | 17806.15 | 7984.82 | 4.97 | 72 |
